# Momentum transfer on impact damping by liquid crystalline elastomers

**DOI:** 10.1038/s41598-023-37215-9

**Published:** 2023-06-20

**Authors:** Hongye Guo, Andrew Terentjev, Mohand O. Saed, Eugene M. Terentjev

**Affiliations:** 1 Cavendish Laboratory, University of Cambridge, Cambridge, CB3 0HE UK; 2 Cambridge Smart Plastics Ltd, 18 Hurrell Road, Cambridge, CB4 3RH UK

**Keywords:** Applied physics, Mechanical engineering

## Abstract

The effect of elastomeric damping pads, softening the collision of hard objects, is investigated comparing the reference silicone elastomer and the polydomain nematic liquid crystalline elastomer, which has a far superior internal dissipation mechanism. We specifically focus not just on the energy dissipation, but also on the momentum conservation and transfer during the collision, because the latter determines the force exerted on the target and/or the impactor—and it is the force that does the damage during the short time of an impact, while the energy might be dissipated on a much longer time scale. To better assess the momentum transfer, we compare the collision with a very heavy object and the collision with a comparable mass, when some of the impact momentum is retained in the target receding away from the collision. We also propose a method to estimate the optimal thickness of an elastomer damping pad for minimising the energy in impactor rebound. It has been found that thicker pads introduce a large elastic rebound and the optimal thickness is therefore the thinnest possible pad that does not suffer from mechanical failure. We find good agreement between our estimate of the minimal thickness of the elastomer before the puncture through occurs and the experimental observations.

## Introduction

Since the beginning of human history, people have been studying and utilising mechanical damping in a variety of materials for suppressing vibration and impact protection. Today, vibration and impact damping extends the useful life of machines or alleviates pain in body implants or in sports kit. Damping materials can be seen in almost every industry: aerospace, automotive, oil rigging, right through to the most delicate electronic devices.

There are many studies in various practical cases, a good example being the study of mouth-guard dental protection: Maeda et al.^1^ have examined the role of pad structure and thickness on impact energy dissipated, while Bochnig et al.^2^ did the comparative study monitoring the acceleration and impact force. Another example of comparative studies, in footwear shock absorption^3,4^, reports and discusses the damping properties of rubber and elastomer pads, at different pad thickness, but also not making a clear distinction between the energy dissipation and the impact force. In this paper, we are trying to reach some clarity about the mechanics and physics at play, pointing out that in different test settings or applications, the different aspects of damping become more relevant. Our goal was to thoroughly characterise the damping properties of an example material that we are familiar with, and often claimed to be a superior damping elastomers, in all practical dimensions, so as to ascertain the most important factors one should consider, and ensure none are missed in studies moving forwards. This is limited not just to peak forces or proxies thereof, but assessing mass and momentum conservation, and discussing the forces on both impactor and impacted surfaces.

The damping phenomenon is caused by different combinations of various fundamental physical mechanisms in different systems. Ultimately, it depends on the intrinsic viscosity in a material that leads to internal energy dissipation into heat^5^. Traditionally, there are two approaches to damping: one based on forming collapsible structures in the material (foams being the most obvious example), the other on the intrinsic viscoelasticity of polymeric materials. Within the latter, the damping devices based on natural rubber, or synthetic rubber such as silicone or polyurethane, are very common in industrial and consumer settings. Although foam-based collapsible damping systems often provide an almost perfect result, absorbing and dissipating all or most of the impact or vibration, in many settings the mechanical strength, stability, or sealing effect of a solid material is required. When the damping efficiency is measured per unit mass of the material or volume, foams and other porous structures are at a disadvantage. Another disadvantage typical in foams is the single-use nature of their structures (losing that damping response once compacted).

Viscoelasticity of polymers and networks is frequently assessed via the linear response modulus, e.g. $G'(\omega ) + i\, G'' (\omega )$, with the loss factor $\tan \delta (\omega )=G''/G'$ as the universal comparative measure of mechanical dissipation at a given frequency^6^. This is a valid and relevant parameter to compare different materials even though it is only applicable in the linear deformation regime, i.e. at vanishing small strains. A ‘high’ $\tan \delta$ in various rubbery network is usually in the range 0.1–0.2, but it could increase as the crosslinking density of a gel diminishes (so $G'$ drops), and it has a characteristic peak increase at the dynamic glass transition when $\tan \delta$ could reach 1.5 or even 2. One of the modern approaches to increasing damping capacity of a material is to use chemical modifications to bring the glass transition (with its peaked $\tan \delta$) into the temperature, or the frequency range of where the damping is needed^5^. For instance, Kuraray products follow this principle in thermoplastic styrene-butadiene elastomers^7^. Unfortunately, in this way one cannot achieve high dissipation outside the narrow region of glass transition. The other modern approach to market-leading damping elastomers is to disperse liquid component into the elastomer matrix, in biomimetic fashion: the principle used in Sorbothane polyurethane composites^8^. The secret of both these approaches is to introduce internal friction through localized viscous flow in the network, however, both introduce significant disadvantages in terms of mechanical properties or stability at high temperatures. Perhaps this is why they are rarely seen in practical applications and in dental and footwear cases, the ordinary rubbers with $\tan \delta$ up to 0.4 are already considered very good dampers^1,4^.

**Figure 1:**
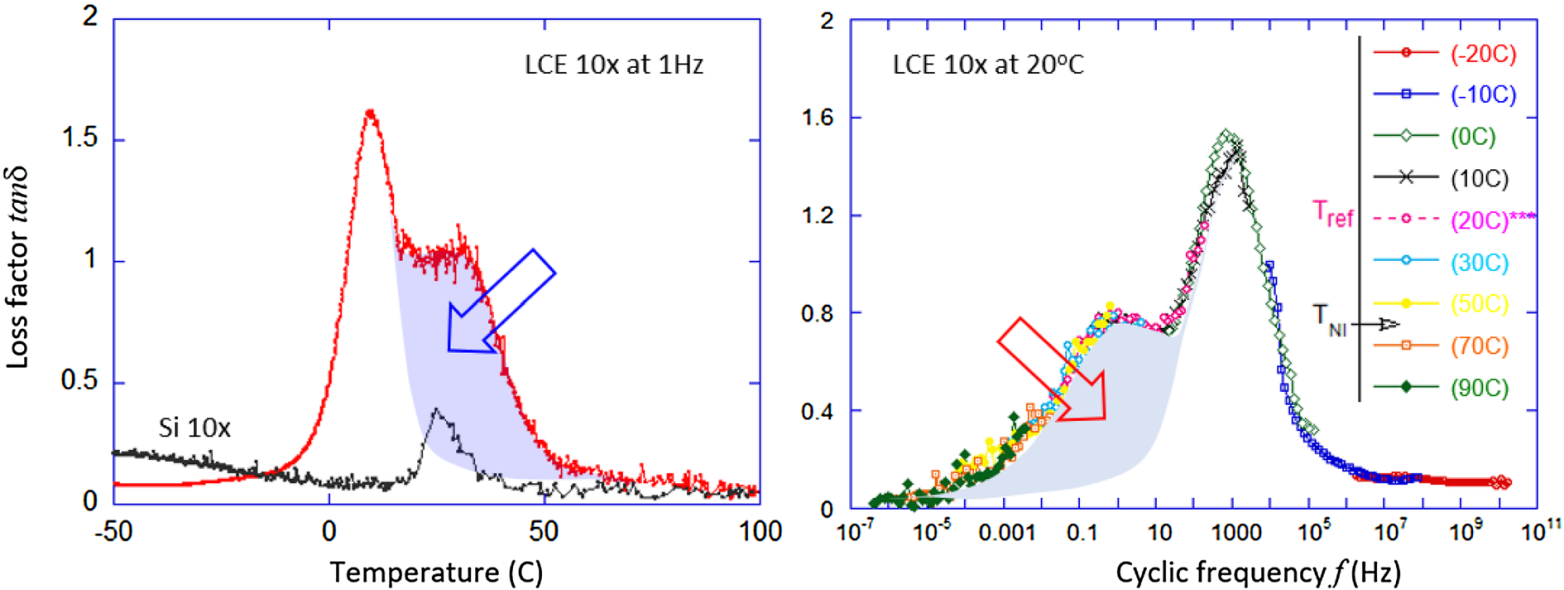
The two representations of loss, expressed by $\tan \delta$ in the linear viscoelastic regime. (**a**) The loss factor as function of temperature, for a 10% crosslinked main-chain LCE at 1Hz^18,21,22^. In addition, this plot shows the loss factor $\tan \delta$ for the 10%-crosslinked silicone elastomer, for reference. (**b**) The Master Curve for the loss factor at a reference temperature of 20 $^\circ$C for the same LCE material, showing the broad range of scaled frequency covered by the time-temperature superposition^6,19,23^. In both plots, the arrow points at the shaded region of enhanced damping in the nematic LCE phase.

With the discovery of nematic liquid crystal elastomers (LCEs), a new candidate in this field of smart polymeric materials that have enhanced viscoelastic dissipation has appeared. The first LCEs were side-chain LCE based on polysiloxane^9^, and the subsequent studies have discovered the anomalous damping of vibrations in these materials^10^. The current understanding of the mechanism for additional dissipation in LCE^11,12^ is based on the unique microscopic network structure of anisotropic chains that allow rotation of local nematic director axis in response to local shear, leading to an additional rotational viscosity route to energy loss. The general theoretical understanding of viscosity and energy dissipation requires a process of overcoming energy barriers by moving particles on the microscopic scale; mostly such barriers arise from intermolecular pair interactions and manifest in the pair correlation functions^13^. The rotation of rod-like mesogenic molecules in the isotropic phase does not meet any energy barriers—in contrast to the nematic phase, when the rotation of mesogenic units must overcome the orientational mean field (proportional to the nematic order parameter)^14^. This provides an additional route to viscous dissipation, not present in any isotropic viscoelastic material, which is emphasized by the plots of loss factor $\tan \delta$ in Fig. 1. The plot of temperature dependence, Fig. 1a also shows the loss factor for the reference elastomer we use in this work: the 10%-crosslinked silicone rubber, chosen to have a similar modulus to the LCE. There is a slight confusion in the literature, frequently attributing the enhanced dissipation of nematic LCE to the effect of ‘soft elasticity’ (or deformation modes without elastic resistance)^15^; in fact, the two are completely separate manifestations of the orientational anisotropy in LCE—soft elasticity plateau arising due to the polymer backbone anisotropy altering the macroscopic sample shape, while rotational viscosity reflects energy dispersal on mesogen rotation against the nematic mean field (which is, of course, directly proportional to chain anisotropy).

This orientational degree of freedom and its associated added dissipation make LCEs a new class of damping materials with a superior performance in dissipation of energy, as demonstrated in the literature over the last 20 years^10,16–20^. The vibration energy dissipation in LCE materials is much higher compared to most polymeric systems, when the broad temperature and frequency range of the effect is taken into account. The enhanced LCE damping is spread over a broad temperature range of the nematic phase (from the glass transition to the isotropic phase, often 80–90 degrees wide range) and a broad frequency range (the Master Curve analysis in Ref.^19^, Fig. 1b, suggests $\tan \delta > 0.8$ from 0.1 Hz to 10 kHz). This is especially important if one is interested in the impact damping, when the short-pulse collision excites a broad range of internal frequencies. In fact, the two recent studies^18,19^ offer a good exposure of LCE damping and energy dissipation in impact settings, which we will explore in this paper.

However, the other aspect of collision impact has received much less attention in the literature: the momentum must be conserved in any such collision, and the dynamics of momentum transfer could lead to a very high force exerted both on the target and the impactor. The energy of the impact may be dissipated almost completely, but the severe damage due to this short pulse of force is still a critical factor in the process. Designers are familiar with this issue, which is why one finds all sorts of extended ‘crumpling zones’ in collision-protection systems, all aiming to extend the time of the momentum transfer and thus diminish the exerted force. This paper focuses on this issue and makes a study of momentum transfer in different collision settings, alongside the energy dissipation analysis. To emphasize: here we do not aim to compare the merits of different damping materials, but use two characteristic and well-studied systems (a super-damping LCE and a silicone isotropic elastomer of comparable mechanical strength) to investigate the effects of momentum transfer, and the impact force generated in the target.

Although the basic theory of energy and momentum conservation is something most readers of this journal have studied in high school, it is worth repeating its steps to highlight the effect of the mechanical energy loss. Consider the initially stationary target of mass *M*, which is hit by the pendulum impactor of mass *m* moving at a speed $v_0=\sqrt{2gh_0}$, where $h_0$ is the elevation of the pendulum before release, an easily measurable parameter. In the pendulum, the momentum exchange takes place along the horizontal axis, while in the simple drop test all the momentum exchange is along the vertical axis. The scalar energy balance includes the mechanical loss: $E_{0}=E_{1}+E_{2}+\text {``Loss''}$, while the vector momentum balance must be maintained exactly: $\textbf{P}_{0}=\textbf{P}_{1}+\textbf{P}_{2}$ along the horizontal axis at the pendulum perigee. Importantly, the sign of $\textbf{P}_{2}$ in the momentum balance needs to be observed carefully, as in some cases the impactor bounces back, while in others in travels with the target (see Supplementary Videos). Explicitly, the two conditions take the form:

1 $$\begin{aligned}{} & mgh_0=\frac{1}{2}Mu^{2}+\frac{1}{2}mv^{2}+\text {``Loss''}, \nonumber \\ & m\sqrt{2gh_0}=Mu\pm mv. \end{aligned}$$

There are three unknowns in these two equations: the speed of the impactor *v* after collision, the acquired speed of the target bar *u*, and the “Loss”. But if we directly measure one or both of the final velocities *u*,*v* then we can determine the energy loss exactly (and also verify that the momentum is conserved). In our experiments, we determine *u*, *v* from the final elevation using the same energy-conservation relation $v=\sqrt{2gh}$.It may seem that the energy equation alone gives this information, but it is important that the momentum balance is observed exactly, which imposes a constraint on the amount of loss. To present the conclusions graphically, we define the loss fraction (the square of restitution coefficient) $R=\text {``Loss''}/mgh_0$, work with the mass ratio *M*/*m*, and express all velocities as ratios to the initial impact speed: $u/\sqrt{2gh_0}$ and $v/\sqrt{2gh_0}$. With these definitions in place, the solutions for the final velocities *u*,*v* take the form:

2 $$\begin{aligned}&\frac{u}{\sqrt{2gh_0}} =\frac{1}{1+M/m}+\frac{\sqrt{M/m-(1+M/m)R}}{\sqrt{M/m} (1+M/m) )}; \nonumber \\&\frac{v}{\sqrt{2gh_0}} = -\frac{1}{1+M/m}+\frac{\sqrt{M/m}\sqrt{M/m-(1+M/m)R}}{1+M/m}. \end{aligned}$$

**Figure 2:**
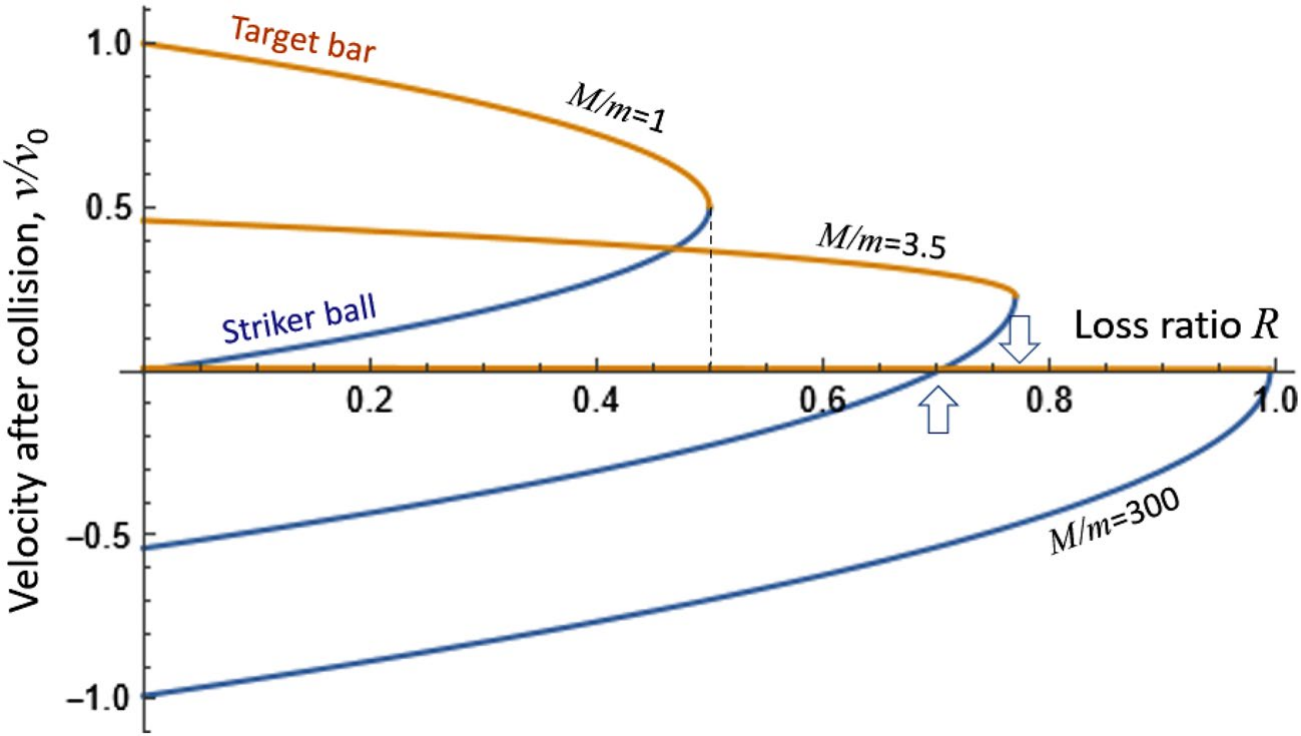
The relative post-impact velocity $v/v_0$ for both the impactor (blue) and the target (orange) plotted against the loss ratio *R*. The three curves corresponds to three mass ratios of $M/m=$1, 3.5 and 300 (modelling infinity). At $M/m = 3.5$, the two arrows point out its theoretical maximum loss ratio, as well as the loss ratio at which the impactor would end up stationary. For the Newton’s Cradle case, the maximum possible loss ratio is $R=0.5$.

When the target object is placed against a solid wall or on a solid floor as in the drop test, it could be viewed as effectively colliding with Earth where $M/m=\infty$, while in our pendulum experiment we have $M/m=3.53$. The case of $M/m=1$ is the familiar Newton’s Cradle case. The graphs in Fig. 2 show the velocities of the two bodies immediately after impact, for these three values of *M*/*m*.

In our study, a generic nematic LCE material was compared with an isotropic silicone elastomer of similar crosslinking density and mechanical strength. For reference, the ”bare” state (metal on metal or metal on granite collision) was also studied. Three damping settings were examined: First, in a simple drop test ($M/m = \infty$) we recorded both the energy balance and the vertical momentum transfer, via the amount of impactor rebound. Then, in order to exactly measure the impact effect on the target, we used a modified Hopkinson-style bar device, where the impact ball at the end of a pendulum hits a long (heavy) target bar rested against a solid wall, with the effective mass ratio still $M/m = \infty$. In this test, we recorded the acoustic pulse propagating along the bar, and were able to calibrate it into the impact force from the momentum transfer analysis. Finally, we studied a simple pendulum set-up where the impactor ball collides with the target bar, both suspended by nylon cords. In this case, the mass ratio of the target to the impactor was $M/m = 3.53$, and the ability of the target to recede away from the collision has reduced the amount of energy loss incurred.

One can see in Fig. 2 that for an elastic collision ($R=0$) the impactor would bounce back at the highest speed (reflected in the negative values in the plot), except at $M/m=1$ when the impactor stays stationary, passing all of its kinetic energy to the target, as in the familiar Newton’s Cradle toy. In the pendulum with $M/m=3.5$, at a value of $R=0.71$, the impactor would remain stationary after the collision. Our observations with the 2 mm silicone pad revealed a loss ratio of almost 70%, and indeed the impactor bounced very little. With the LCE damping pad, we obtained the maximum possible loss ratio in this experiment (for the given *M*/*m* ratio), as marked in the plot by the arrow at $R=0.76$. The reason why there is a limit on the observable loss is due to the momentum conservation: as the target bar is accelerating away from the collision, and the impact ball decelerating, at some point the two velocities *u*, *v* would match (as seen in the contact apex of the two curves in the plot), and no more mechanical deformation would occur to be dissipated as loss. For the case of two equal masses, $M/m=1$, the maximum possible dissipation is just $R=0.5$. Only when $M=\infty$ (so that $u=0$ in the target) could the impact ball dissipate all of its energy and achieve $R\rightarrow$100% if the damping material and structure would allow that. In such an ultimate case, the impact ball would stay stationary in front of the target after the collision.

In carrying out this study, we have also examined the effect of the damping pad thickness. It might seem that the thicker the protective pad, the less ‘damage’ the collision would cause. This is true for the protection of the target, but when considering the impactor this statement does not give the full picture. Damping with collapsing foams may favor thicker pads in all scenarios, however, with solid elastomers it is far from the case. The reason is that the elastic energy is stored in the elastomer during the impact, and then released back affecting both the target and the impactor. The minimisation of rebound energy in the impactor (and the total impulse it recieves) is achieved for the thinnest protective pads, as long as they are not broken through during the collision.

## Experimental details

### Materials

The LCE damping pads were based on the now-standard synthesis^24,25^ using commercial 4-(3-acryloyloxypropyloxy)benzoic acid 2-methyl-1,4-phenylene ester di-acrylate mesogens (RM257), obtained from Daken Chemical Ltd. 2,2$^\prime$-(ethylenedioxy) diethanethiol spacers (EDDT), and pentaerythritol tetrakis(3-mercaptopropionate) 4-functional crosslinker (PETMP) were used to form the network of main-chain nematic polymer, adding butylated hydroxytoluene inhibitor (BHT) to prevent acrylate homopolymerisation, and Dipropylamine catalyst (DPA) for the Michael addition thiol-acrylate click reaction. All these chemicals were obtained from Sigma Merck.

LCE damping pads used in this study were made in a solvent-free environment to better control their thickness. All LCE materials were used in their natural polydomain state^10,15^, which we believe further enhances the internal damping. The composition given below was used in many other studies^18,19,21^ and would result in a 10% cross-linking density. For every 10 mmol of RM257, 9 mmol of EDDT, 0.5 mmol of PETMP and 2 wt% BHT were added into the same container. All precursors were melted and mixed, assisted by gentle heating until a clear homogeneous liquid is formed. The mixture was then placed under vacuum at 70$^\circ$ for 5 min for de-gassing. It was then allowed to cool down to roughly 50$^\circ$ before adding 0.5 wt% DPA catalyst, immediately followed by careful mixing with a spatula. This step is especially critical as one must be quick to disperse the DPA catalyst without generating more air bubbles in the already de-gassed reaction mixture. Given the fast curing process, de-gassing after adding the catalyst was impossible. The reaction mixture was then poured into prepared Teflon moulds with varying shape and thicknesses, and allowed to cure at room temperature overnight. To complete the crosslinking and ensure no unreacted moieties remain, the moulds were then brought to 80 $^\circ$C oven for another 24 h.

The control damping pads made from an isotropic silicone rubber were prepared using the Sylgard 184 preparation (from Dow Corning). The two-component mixture was prepared using 10 wt% of hardener, to approximately replicate the crosslinking density of the LCE. After mixing and de-gaSsing, the viscous compound was poured into Teflon moulds and allowed to cure at room temperature overnight. To complete the crosslinking and ensure no unreacted moieties remain, the moulds were then brought to 80 $^\circ$C oven for another 24 h.

To be clear, both materials used for contrast have their glass transitions much lower than the ambient measurement temperature ($T_g (\textrm{LCE}) = 0\;^\circ$C, $T_g (\textrm{Si}) = -60\; ^\circ$C) and so none of the glassy dissipative dynamics plays any role here. With all materials, we had damping pads match the dimension of target rods, of 30 mm diameter, and thickness varying from under −1 mm to almost 20 mm.

### Drop test set-up

Drop tests were performed by dropping a stainless steel ball (25 mm diameter) from a height of 3.2 m above a heavy anti-vibration granite base (50 $\times$ 50 cm, 50 mm thick) to achieve an initial impact energy of ca. 2 J. The energy measurements of the impactor ball rebounding after impact were obtained by analysing camera captured footages at 60 fps. See the Supporting Video 1 for the illustration of this test.

### Hopkinson-style ball-bar set-up

A traditional split-Hopkinson pressure bar (also known as Kolsky bar) is composed of three axially aligned metal rods: the striker, incident and transmission bars. Normally, the striker bar is launched by compressed air through a gun barrel. The testing specimen is situated in between (and in contact with) the incident and transmission bars. The impact generates a compression wave that travels along the incident bar—specimen—transmission bar assembly and is detected by strain gauges mounted on both bars. By analysing the voltages signals from the gauges, one can obtain the incident, reflected and transmitted stress wave profiles^26^.

Using the same principle, a Hopkinson-style testing platform was built to record the impulse signals in the target bar, protected with different damping pads. Unlike the traditional set-up, here a stainless impactor ball (50 mm diameter) was used instead of a bar with a flat impact surface, in order to generate more shear stress in the pads. This serves to better excite director rotations in LCE damping pads. The ball was suspended by nylon cord from a frame and raised to 0.84 m (providing the impact energy of ca. 4.5 J). Upon release, the striker ball would fall and collide with damping pads placed in front of a long brass bar (30 $\times$ 30 $\times$ 1500 mm) resting against a solid wall. To record the impulse signal generated by this impact, four strain gauges (AFP-500-090, from Kulite) were mounted close to the impact surface and were connected to an oscilloscope (Analog Discovery Pro). The four gauges were configured to from a ‘full Wheatstone bridge’ circuit with two gauges rotated by 90$^\circ$. To trigger the oscilloscope, a pin laser module (APCD-650-01-C2 ) and a photodiode (also connected to the same oscilloscope) were placed in such a way that the striker ball would block the laser moments before impact: the drop in the photodiode output would trigger the data acquisition process. Periodic noises and offset in the signal were removed by subtracting the baseline. The trajectories of the impactor ball were filmed at 60 fps and analysed to determine its energy and momentum after the collision. See the Supporting Video 2 for the illustration of this test.

### Simple pendulum set-up

A simple pendulum set-up was built on a frame, with a mild steel target bar (300 $\times$ 30 $\times$ 30 mm) and a stainless steel impact ball (25 mm radius). Eye-bolts were mounted onto both entities, one for the impact ball and two for the target bar (one at each end, parallel to the impact surface, to minimise rotational movement). Through these eye-bolts, the ball and bar were suspended by nylon cords with the top ends clamped on a frame. Gratings sheets were placed behind to aid the frame by frame analysis of footages captured at 60 fps. See the Supporting Video 3 for the illustration of this test.

## The drop impact test

**Figure 3:**
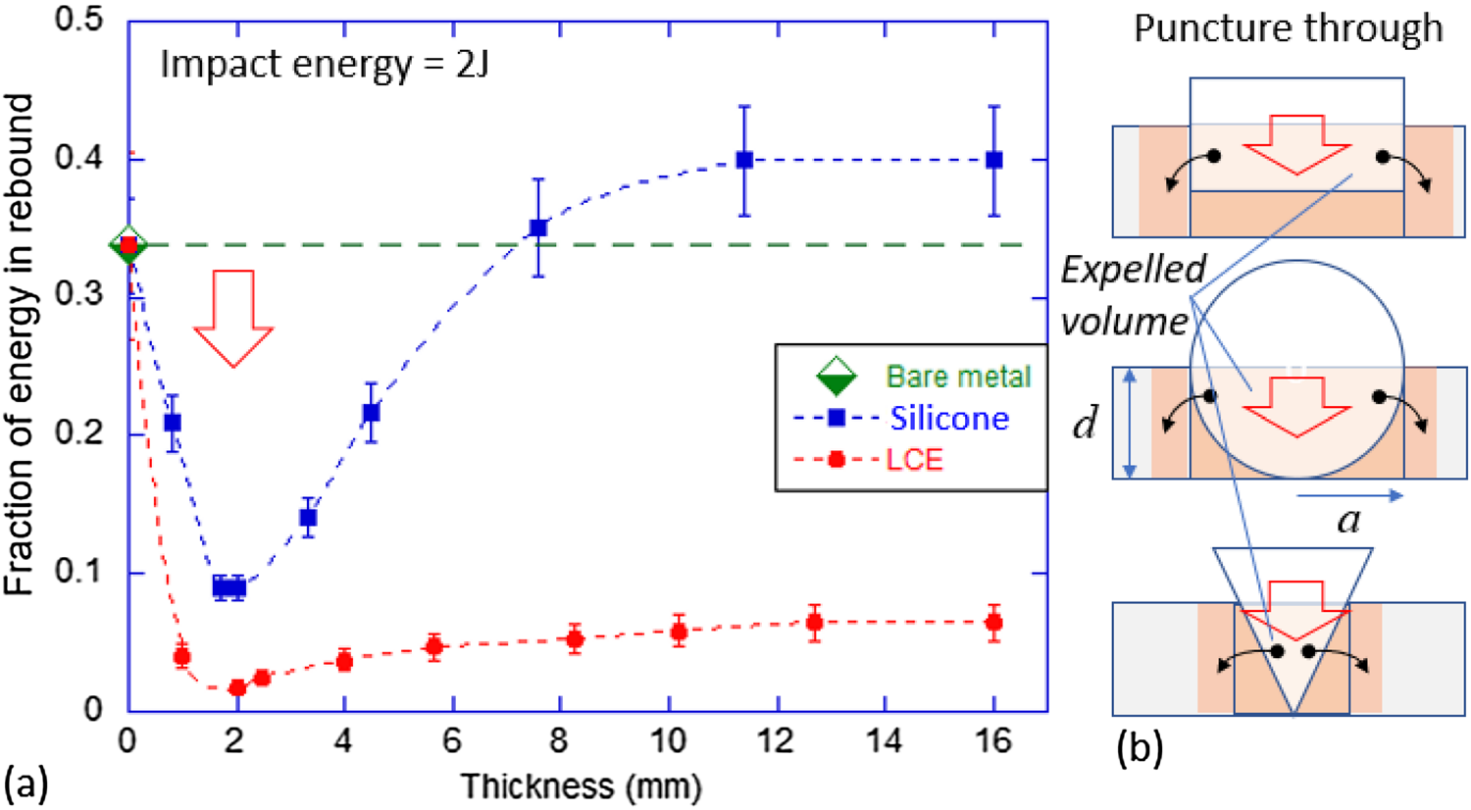
(**a**) Fraction of energy in rebound plotted against damping pad thickness for silicone (blue) and LCE (red) in the vertical bounce of granite plate, with the initial $E=$2 J. The dashed line represents the rebound of bare metal on granite. (**b**) Sketches of the geometry of puncture estimate, illustrating the ‘affected volume’ as a sum of the cylindrical shape under the impactor plus the volume displaced by the impactor (the calculation depending on the impactor geometry). Error bars on our data, arising from multiple repeated tests, are shown in the plot.

This test is almost trivial in design, in our case with the steel ball ($m=64$ g and diameter 25 mm) released from a controlled height $h_0$ and hitting the thick anti-vibration granite plate. To test the effect of damping by elastomers, we used silicone and LCE pads of different thickness, so that the dropping ball hit a fresh pad every time. We monitored the height of the impactor ball rebound, which gives us the take-off velocity $v=\sqrt{2gh}$. We have studied this rebound for various different initial elevations for the completeness of this picture, but here for the sake of clarity we only present the case of a specific $h_0=3.2$ m and the initial energy of impact $E_0 = 2$ J. For the bare metal collision, the loss ratio of the rebound energy to the initial one $R=0.34$ (which means the restitution coefficient is ca. 0.6). Figure 3a shows the loss ratio in this context, for different thickness values of damping pads.

It is immediately clear that thinner pads cause greater energy loss, except when the thickness fell below 2 mm in this test—thinner pads were punctured through and destroyed by the impact (see the illustrative images in Supporting Information). It is, therefore, important to estimate the minimal thickness of the protective elastomer pad that is expected to survive the impact, since this would be the optimal damping outcome to achieve the lowest energy in rebound. For silicone, this optimal loss ratio represents about 90% energy loss, while in the LCE the loss ratio is above 98%. Sketches in Fig. 3b illustrate how we estimate the minimal thickness before puncture through. Obviously, the outcome depends on the shape of the impactor, as the sketches point out. The aim is to estimate the volume of the elastomer material affected by the puncture, which is shaded in the sketch: this volume multiplied by the characteristic energy density the material can tolerate (given by its Young modulus $Y \sim 3$ MPa in our case) would be equal to the impact energy at the critical point of first puncture. As we focus on the spherical impactor of radius $r = 12.5$ mm in our case, we need the radius of the spherical cap of height *d*, which is $a=\sqrt{2rd-d^2}$, and then take the affected volume of the material *V*(*d*) as the sum of cylindrical volume of radius *a* and the expelled volume of spherical cap. We then just need to solve the equation $E_0=V(d)\cdot Y$ to estimate the critical thickness *d* for puncture. This is a simple cubic equation with just one real root, and the estimate for $E_0=2$ J is remarkably accurate: $d \approx 2.1$ mm.

At the optimal damping pad thickness, we record the very high loss ratio for the LCE: $R=98.3$% (and $R=91$% for silicone). We now turn to the Hopkinson-style bar test to see what forces propagate into the target on such an impact, something that was not possible to assess in the drop test.

## The Hopkinson-style bar impact test

Comparing to the traditional Hopkinson split pressure bar experiment^26^, our modified set-up uses a solid ball ($m=564$ g and diameter 50 mm) suspended from a pendulum as the impactor. Instead of the flat-to-flat surface impact, the impactor ball can generate significant shear stress and promote the rotational motion of mesogen cores in the LCE damping pad. The discussion of the merits of non-flat impactors to avoid purely compressional deformations could be found in Ref.^19^.

**Figure 4:**
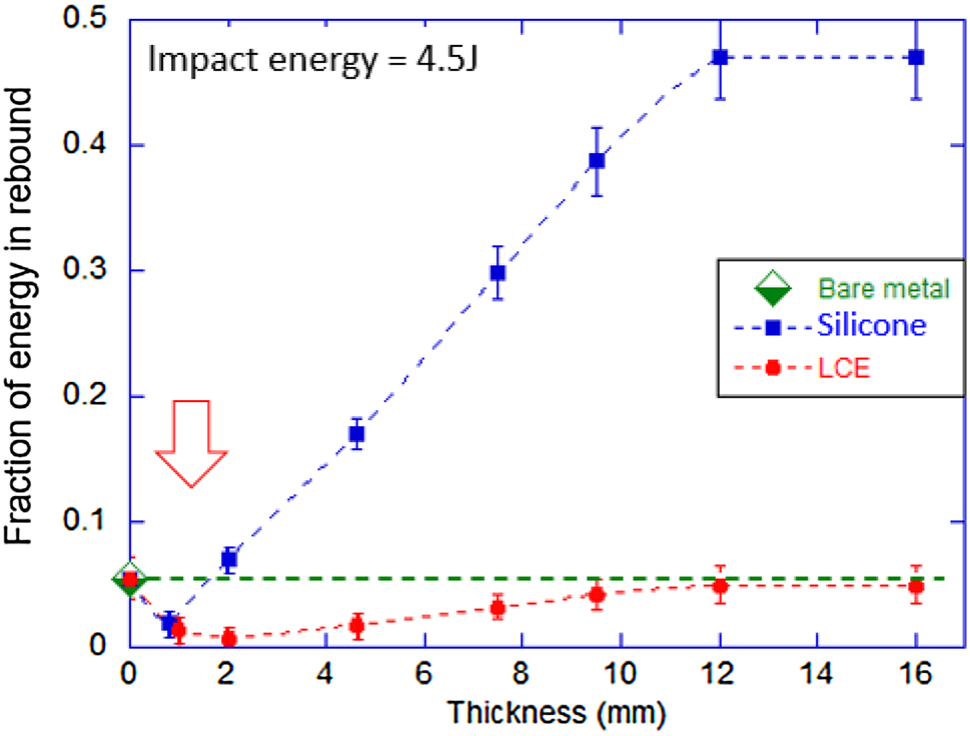
Fraction of energy in rebound plotted against damping pad thickness for silicone (blue) and LCE (red) in the impactor ball rebound from the Hopkinson-style bar resting against solid wall, with the initial $E=$4.5 J. The dashed line represents the rebound of bare metal on metal. Error bars on our data, arising from multiple repeated tests, are shown in the plot.

In the study of the large Hopkinson-style target bar resting against solid wall (essentially the collision with Earth, $M=\infty , \ u=0$), we can again measure the rebound velocity *v* from the video recording of the rebound, and thus calculate the loss ratio exactly. Figure 4 shows the result similar to that in Fig. 3a, that is, the loss ratio for different thickness values of damping pads, for the impact energy $E_0=4.5$ J (much higher than in the drop test before). We see exactly the same trend, with thinner pads providing much better reduction of the impactor energy, until the puncture through happens. In this case, with the ball radius $r=25$ mm and higher impact energy, the estimate of the critical thickness gives $d \approx 1.9$ mm, again surprisingly accurately representing the observation.

At the optimal damping pad thickness, we record the loss ratio for LCE: $R=99.2$%, and for silicone: $R=98$% (although both need to be put in context of the restitution of the bare metal collision, $R=94$%). Much of this ‘loss’ represents the energy passed into the target bar, which is what this particular test aims to capture. These results at a higher impact energy emphasise another clear fact: while both elastomer materials dissipate impact energy, LCE does not store much of the elastic energy in both its compression and return to original dimensions. This can be seen by the modest damping effect seen even at maximum thicknesses tested. In contrast, the elasticity of our silicone sample on return to its original shape after collision releases a substantial energy back into the impactor. It is therefore a poor damping material in terms of protecting the impactor.

There are multiple routes of energy loss in this collision: some energy transfers into heat in the elastomer pad, some is gone into sound waves in the surrounding air, and one part of the ‘lost’ energy is transmitted into the target bar as an elastic impact wave. In this experiment, we monitor this elastic wave with a set of calibrated strain gauges mounted on the bar (as in the classical Hopkinson bar design), and record the pulse signal as the wave propagates along the bar. Normally, it is very difficult to scale (calibrate) this signal to convert it into the force acting on the target bar, for the surface-mounted sensors measure a trace of the complicated geometry of acoustic waves generated in the target bar. Most of the literature^18,26^ describes methods that rely on calibration of sensors and electronics, while the biggest uncertainty lies in the way the local strain is picked up on the surface of the bar (and a question of how well this reflects the full acoustic wave structure in the bulk). However, in our case, there is a remarkably easy way to achieve a reliable calibration, due to our monitoring of the momentum transfer. After measuring the rebound speed *v*, we know exactly the impulse $\Delta P$ delivered to the target bar. The Wheatstone circuit constructed by four strain gauges provided voltage signals which are proportional to the strain and thus the force acting upon the target bar. Thus, the following can be written:

**Figure 5:**
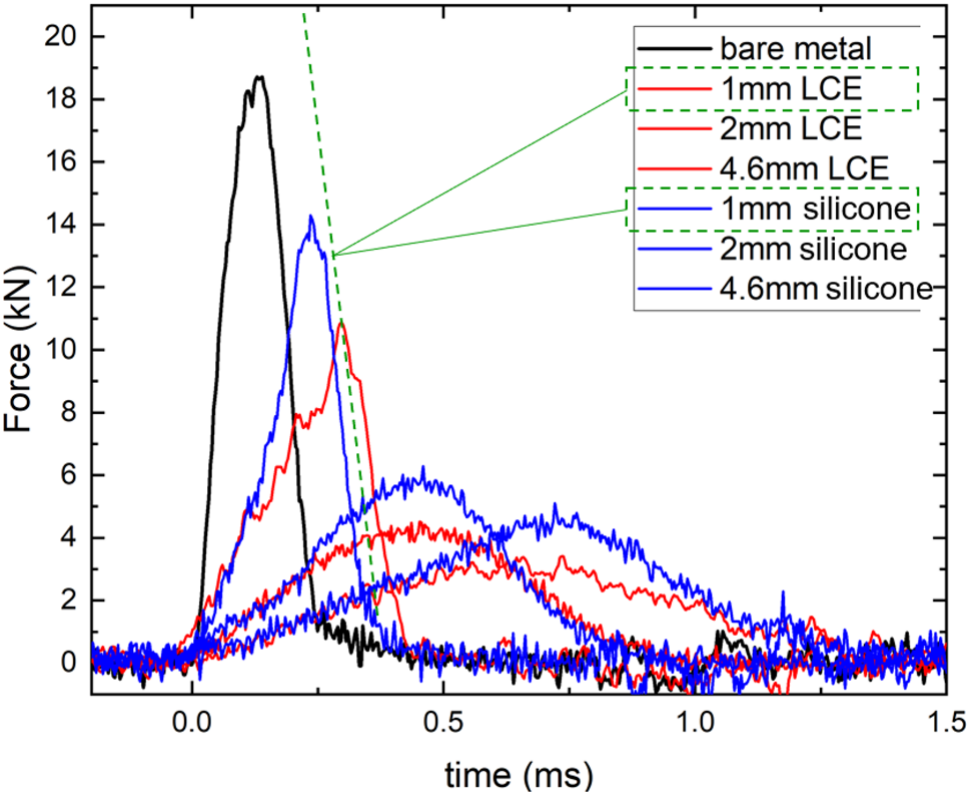
Impulse peaks in the time domain for bare metal (black), silicone (blue) and LCE (red). The effect of damping pad thickness is evident: thicker pads spread the width of the peak over the time that the compression wave travels across the pad. In all cases, the LCE damping pad performs better than the isotropic silicone elastomer of similar crosslinking density and modulus. The dashed line marks the peak slope at the receding edge of the pulse for the two (1 mm) damping pads that were punctured through by the collision.

3 $$\begin{aligned} \Delta P =m(\sqrt{2gh_0}+v)=\int F(t)dt=\alpha \int V(t)dt, \end{aligned}$$

where $\alpha$ is the calibrating factor. To demonstrate the scaling procedure, let us take the bare metal-to-metal setting as an example: The overall momentum change of the impactor ball was found to be $\Delta P = 2.74$ Ns, and the total area under the voltage peak was calculated to be $\int V(t)dt = 2 \times 10^{-5}$ Vs. Therefore, the calibrating factor $\alpha$ can be obtained as $\alpha = 137,252$ N/V. Figure 5 shows the impulse (force vs time) for the three damping settings: with the ball striking the bare metal bar, with silicone damping pads, and with LCE damping pads.

**Figure 6:**
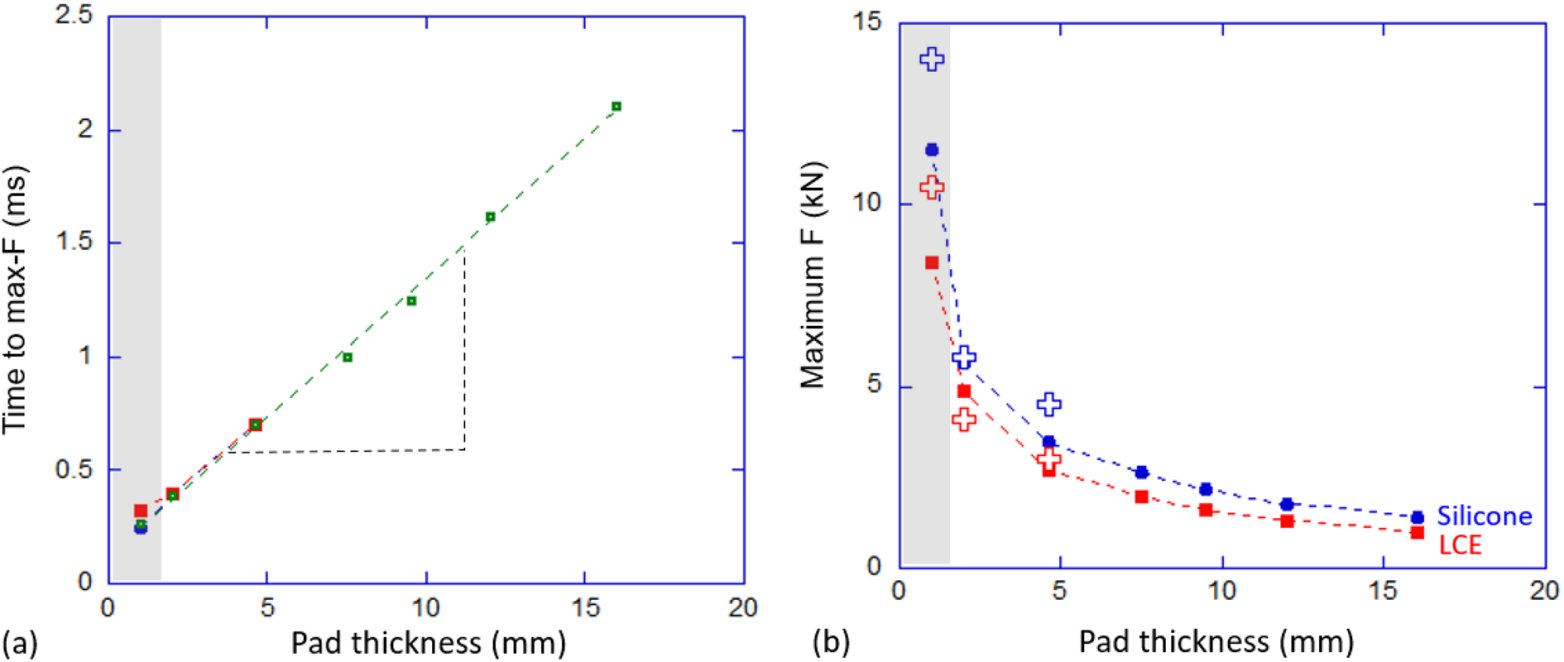
(**a**) Measured time of the front of the impulse peak. The data plotted in Fig. 5 are shown separately, with colors matching the legend of Fig. 5; The green-color data points are from our separate measurements with thicker pads, not shown in Fig. 5 to preserve clarity of illustration. To reiterate the point in the text: these time shifts are the same for LCE and silicone pads. The slope of the line represents the speed of 4 m/s. (**b**) The estimated values of maximum force, predicted by the integral formula (see text). The measured values seen in Fig. 5 are labelled by large cross symbols, which are quite close to the theoretically predicted values. In both plots, the shaded region marks the thin pads punctured through by the impact.

Two things occur as the damping pad thickness increases: First, the maximum forces acting upon the target bar decrease drastically. With no damping medium, the bare metal-to-metal response reached almost 19 kN. This value dropped down to around 3 kN when a 4.6 mm LCE damping pad was inserted. Second, the width of the impulse peak increases. With 2 mm and 4.6 mm damping pads, the impulse responses from LCE and silicone have the width of 0.8 and 1.3 ms, respectively (not surprisingly, the same for both materials, since the speed of the leading compression wave is similar in them). With much thicker damping pads, our strain gauges struggled to distinguish the much weaker peak force from the background noise: the data was hard to present, although one could still discern the width of a broad impulse peak. The Supporting Information gives an example of multiple tests (producing and comparing the degree of overlap of several traces of the impact force as in Fig. 5), illustrating the error due to noise in our setup, which is ca.0.5 kN.

Plotting the measured width of the impulse peak against the pad thickness, Fig. 6a, we obtained an accurate linear variation, with the error (determined from multiple tests) not exceeding 0.05 ms: not worth presenting as error bars in the plot. The slope of this linear dependence, marked in the plot, indicates a constant velocity of propagation of ca. 4 m/s. This was almost exactly the initial speed of the incoming ball (determined from $\sqrt{2gh_0}$), and is much slower than the speed of the compression wave in these elastomers (which is ca. 1500 m/s). This shows that the shape of the impulse peak in Fig. 5, and therefore the maximum force as well as the net impulse $\Delta P$ (which we can measure accurately from the rebound data for all pads), are determined by the evolution of the quasi-equilibrium stress–strain pattern in the elastomer as the striker ball slowly penetrates into the depth of the pad. The pattern itself establishes much faster, on the time scale established by the high speed of sound. Since our pads were all much thinner than the diameter of the impactor ball, we cannot use the classical Hertz solution for the indentation of a sphere into an elastic half-space (which gives the force $F \propto x^{3/2}$)^27^, but the fundamentals remain the same. Clearly, in the much thicker pads, the width of the impulse peak, and the maximum force, would saturate at the values determined by the Hertz solution (although the strain magnitude in the target bar is too low to detect in our setup). We could estimate the maximum force from the simple argument: looking at the curves in Fig. 5 and assigning an approximately Gaussian shape to the impulse peaks, we can integrate the area under each curve, since we know the time duration of each peak. Since we measure this area independently as the impulse $\Delta P$, the estimate gives: $F_{\textrm{max}} = \Delta P/1.253 \Delta t$, the numerical factor arising from the Gaussian integration, which is plotted in Fig. 6b as theoretical prediction, and compared with the three values of $F_{\textrm{max}}$ measured from Fig. 6a. To reiterate: all these points are for the pads much thinner than the Hertz half-space solution requires, that is, when the thickness $d \gg$ ball diameter of 50 mm, where we expect both $\Delta t$ and $F_{\textrm{max}}$ to saturate.

The 1 mm damping pads are below the critical thickness for the puncture through. Here, the response pulse arising from LCE damping pad is slightly wider than that of silicone, which is clearly due to the difference in the incoming slope of the curve. The high-frequency damping in LCE is sufficient to make a difference even at this short time scale, and the peak force getting through the LCE pad is noticeably lower than for silicone. Once the puncture occurs, we are testing the metal on metal contact, and the receding slopes of both thin damping pads are the same as that of the bare metal impact (as illustrated by the dashed line in Fig. 5). In practice, we observed clear holes in both damping pads where the impactor has punctured through. Regardless of the damping pad thickness, LCE resulted in lower maximum force than silicone, as expected for this superior damping material.

We now turn to examine the case of smaller mass ratio, when the target bar is able to recede away from the collision (i.e. speed $u \ne 0$) during the time that takes for the momentum transfer (recall the discussion in Fig. 2).

## Pendulum impact test

In the simple pendulum set-up, the impactor ball ($m=564$ g and diameter 50 mm) was suspended by one nylon cord, while the target bar ($M=1.86$ kg) was suspended by two nylon cords to minimise its rotational movement. The mass ratio of the two bodies was $M/m=3.53$. By adjusting the initial height of the ball, one can examine the impact at different initial momentum/energy (still utilizing the $v=\sqrt{2gh_0}$ relationship at the lowest point of the pendulum swing). By analysing the frames of camera-captured motion, the final positions (heights) of the target bar and the impactor ball were measured, and converted to their momentum and energy immediately after the impact. The results are listed in Table 1.

**Table 1:** Energy *E* (J) and momentum *P* (kg m/s) of the impactor ball and the target bar before and after collisions. All data for 2 mm damping pad thickness. In the last column we show the sum of all momenta (which must be zero in theory); the deviation of $\sum P$ from zero reflects the experimental error of our experiment, which is ca.3% as determined from multiple repeats of each test. The fact that it is consistently negative reflects the inevitable energy loss in the air friction of the incoming ball, and in the nylon cords.

Settings	Before		After					
	$E_0$	$P_0$	$E_{\text {ball}}$	$E_{\text {bar}}$	Loss	$P_{\text {ball}}$	$P_{\text {bar}}$	$\sum P$
Bare metal	4.37	2.16	0.36	2.14	43%	0.62	2.83	− 0.06
	2.91	1.76	0.30	1.49	38%	0.56	2.37	− 0.04
	1.57	1.29	0.21	0.88	31%	0.47	1.81	− 0.05
LCE	4.37	2.16	0.06	0.99	76%	− 0.25	1.93	− 0.06
	2.91	1.76	0.04	0.72	74%	− 0.21	1.64	− 0.09
	1.57	1.29	0.01	0.42	73%	− 0.11	1.25	− 0.06
Silicone	4.37	2.16	0.01	1.45	67%	0.12	2.33	− 0.06
	2.91	1.76	0.01	0.99	66%	0.11	1.93	− 0.04
	1.57	1.29	0.01	0.56	64%	0.08	1.45	− 0.07

First, let us compare all three damping settings at $P_0=2.16$ kg m/s (or impact energy $E_0=4.37$ J). With no damping between the impactor and the target, the bare metal collision resulted in the smallest loss ratio of 43% (corresponding to the restitution coefficient of 0.66); when a 2 mm silicone pad was placed in front of the impact surface, the loss ratio increased to 67%; when a 2 mm LCE damping pad was involved, the loss ratio has reached its theoretical maximum of 76%, as predicted in the analysis of Fig. 2. To further confirm this observation, the same impact experiment was repeated with a 4.6 mm thick LCE pad, and the exact same loss ratio of 76% was obtained. The rebound observations also are of a stark contrast between the bare metal collision (when a high rebound speed was registered), the traditional damping pads of 2 mm thick silicone rubber (when we saw the rebound speed of almost zero), and the anomalous damping of LCE pad of the same 2 mm thickness, when a clear follow-through of the impactor ball was observed indicated by the negative $P_\text {ball}$ values in Table 1.

Next, the collisions in the same damping setting were examined at different initial momentum/energy. In all cases, a lower impact energy directly resulted in a lower loss ratio, although the differences were much more significant in the bare metal impact. Note that in this experiment, the overall energy dissipation had four routes: energy absorption by the damping pads (if present), acoustic waves generated by the impact (in the air), elastic waves in both the ball and the bar, and other instrument losses (air resistances, frame vibration, etc.).

## Conclusion

Although the aim of this work was not to compare the efficiency of damping materials in any great detail, and we have not listed results in other ‘competing’ materials obtained with natural rubber or Sorbothane damping pads to preserve the clear story—we do note that this study reconfirms that LCEs are indeed one of the best viscoelastic damping systems. Our main point here was to compare different regimes of impact damping, and although we could legitimately claim that the LCE damping pad with optimized thickness dissipates over 98% of impact energy, which is by a margin greater than any other damping material, the value itself requires a lot of commentary. For instance, in the pendulum setup we recorded the energy dissipation by LCE of just 76%—but this turns out to be the absolute maximum possible for the given mass ratio of the colliding objects. Understanding of these delicate aspects of inelastic collisions only arises from the analysis of mandatory momentum conservation, which is not often paid attention to in the literature on this subject.

One of these aspects examined here is about ‘which side’ of the collision needs to be protected. One could be interested in the least possible force and energy acting on (or left in) the impacting object, or in reverse, be concerned about the least amount of force and energy passing through into the target object. Inserting a damping elastomer into such a collision is just one of the methods of addressing these questions, while the design of collision conditions (and specifically, the momentum transfer) plays a big role in the outcome, differently on each side.

Another important element, and a conclusion of this study, is the effect of damping pad thickness, which has also been the focus of several practical use-case studies in dental and footwear applications^1,3^. Unlike in damping by collapsible foams or other porous systems, with the internal viscoelastic damping of solid elastomer we find that the thinner the damping pad, the greater the fraction of energy dissipated (which we measure by the restitution, or the loss fraction *R*). The essential limit of this, is when the damping pad is too thin and the impactor punctures through it, destroying both the pad and the energy dissipation mechanism. Here we offered a simple estimate of the minimal elastomer thickness before this puncture through occurs, which should help designing optimal impact damping constructions.

Related to the possible damage of the damping pad on impact, such as with the puncture through, we need to reflect on the possible reversible ‘damage’ to the LCE pad arising due to the local orientational alignment of the material where the local stress far exceeds the threshold of the polydomain-monodomain transition^15^. It is well-studied effect that may appear as local plastic deformation, because (especially in main-chain LCE materials we use here) the metastable aligned texture could be arrested^28^. In the case of localized impact, when the force of impact is too high (in practice: close to the puncture-through limit), a ‘spot damage’ could be left, presumably altering the natural damping conditions in the region. Such a metastable ‘spot damage’ is eliminated by annealing the LCE to its isotropic phase, to reset its natural polydomain structure. However, we also noticed that, since the rate of impact is very short (milliseconds), the softness of the polydomain-monodomain transition was much harder to reach, and in practice we did not obtain the described metastable ‘spot damage’ unless the impact force was very near the breaking limit.

Although this was not our focus here, the better understanding of internal dissipation mechanisms is still needed in this field. Our work has demonstrated clearly that LCE is indeed a superior and perhaps the best damping elastomer available. There is a broad understanding that LCEs are so good in dispersing internal mechanical vibrations due to their internal rotational degrees of freedom. However, further study into how other internal structures, for instance the hydrogen-bonded transient network of polyurethane, or the presence of dopants of silicone oil or glycerol, present in materials like Sorbothane, is called for. The detailed understanding of what actually happens with polymer chains at a high frequency/short time of the collision would be very helpful, and further focused research into these internal mechanisms is needed to inform and guide the design of superior damping elastomers.

## Supplementary Information

Supplementary Information 1.

Supplementary Video 1.

Supplementary Video 2.

Supplementary Video 3.

## Data Availability

All data generated or analysed during this study are included in this published article [and its supplementary information files].
