# Supplementary material for: Momentum transfer on impact damping by liquid crystalline elastomers
Source: Sci Rep. 2023 Jun 20;13:10035. doi: 10.1038/s41598-023-37215-9 (PMC10282006; doi:10.1038/s41598-023-37215-9)

Momentum transfer on impact damping by liquid crystalline elastomers

Hongye Guo, Andrew Terentjev, Mohand O. Saed, Eugene M. Terentjev

SUPPORTING INFORMATION

Supporting Video 1: Drop test

This video shows three drop tests merged together in one sequence – first the metal ball dropping on the bare granite plate, then on the silicone pad of 2mm thickness, and then the LCE pad of the same 2mm thickness (as illustration: we have done the same test for many different thickness pads). In each case, the video allows to measure the rebound height, in the LCE case, the rebound energy is less than 2% of the impact energy.

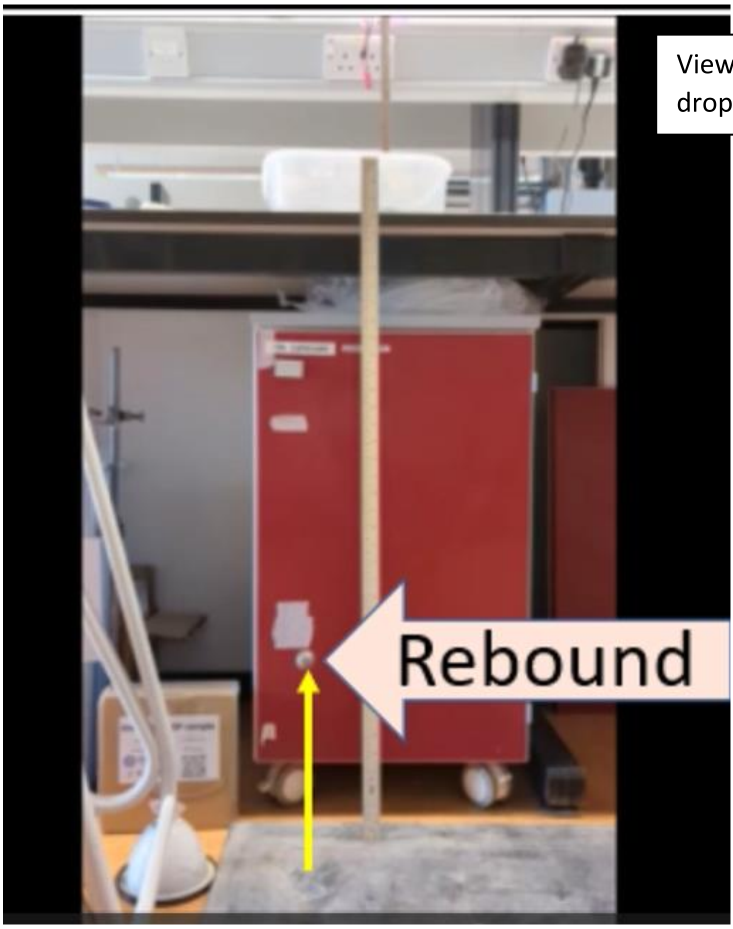

View from the side. The steel ball is dropped from 3.2m height

Supporting Video 2: Hopkinson bar test

This video shows three Hopkison-bar tests merged together in one sequence, for illustration, in all cases using the LCE pad – first the 1mm thickness (which shows puncture through), then 4mm thickness, and then 12mm thickness. In each case, the video allows to measure the rebound distance, from which we calculate the impulse of the collision (since the momentum must be conserved).

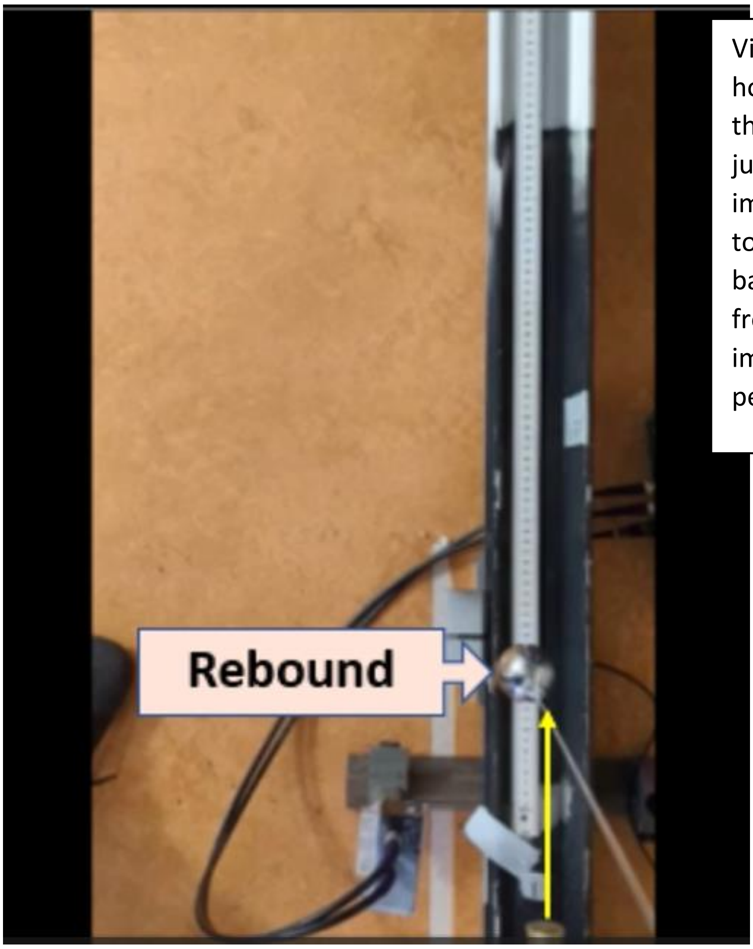

View from the top, along the rail holding the steel bar resting against the solid wall. The face of the bar is just visible at the bottom of the image, the ruler in front of it is used to measure the rebound. The steel ball is suspended on a rigid string from a point directly above the impact point, and dropped in the pendulum motion to hit the target.

Supporting Video 3: Pendulum test of momentum conservation

This video shows three pendulum tests merged together in one sequence – first the metal ball striking the bare metal rod, then on the silicone pad of 2mm thickness, and then the LCE pad of the same 2mm thickness (as illustration: we have done the same test for several initial impact energies). In each case, the video allows to measure the rebound, or in the LCE case – the follow-through.

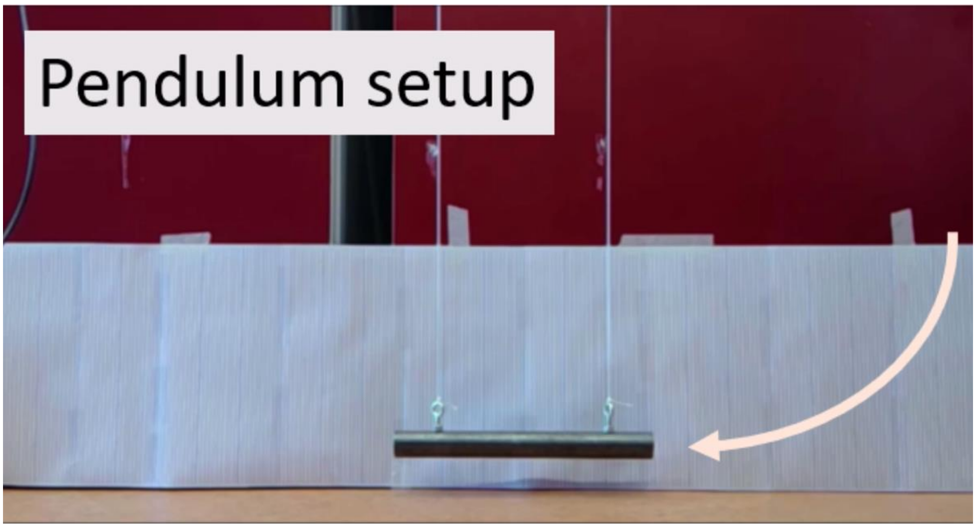

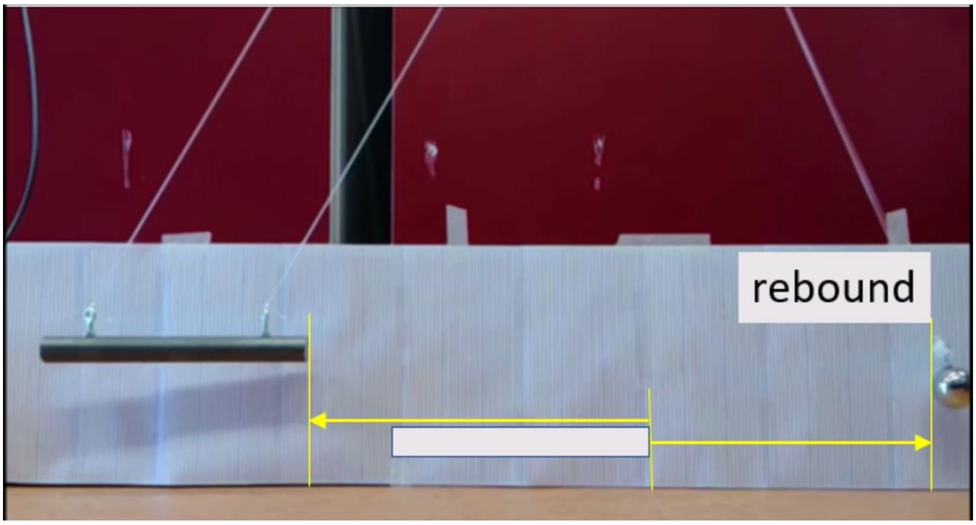

The examples of puncture-through holes in silicone (two holes) and in two different LCE pads, with the scale bar applying to all photos. The different nature of damage is clear, and originates from the pure elastic nature of silicone elastomer (with cracking the failure mode), in contrast to the soft-elastic response of LCE that creates a round 'image' of the impactor wide around the hole/.

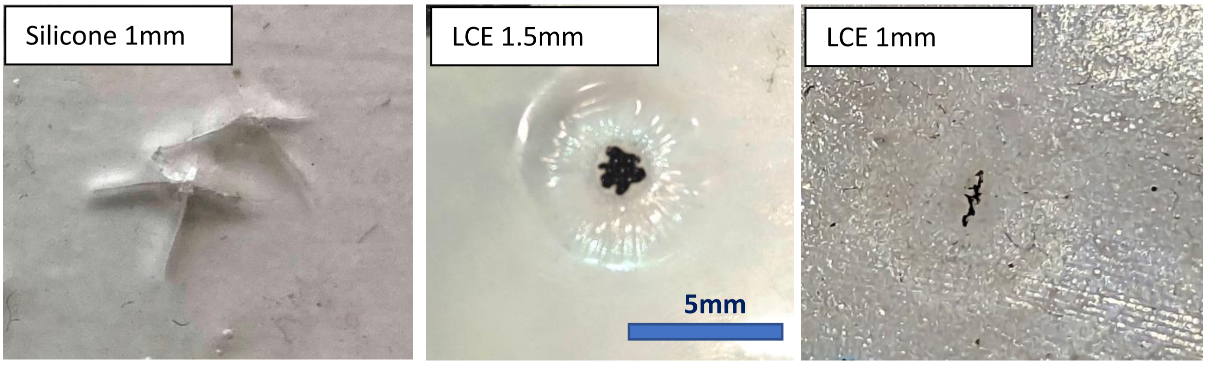

Several repeats of the Hopkinson bar test, measuring the timed trace of the impact force (after the normalization from the separately measured total impulse, as described in the text). This demonstrates the reproducibility of the test and the error due to noise, which is not relative to the value, but ca. 500N in absolute units.

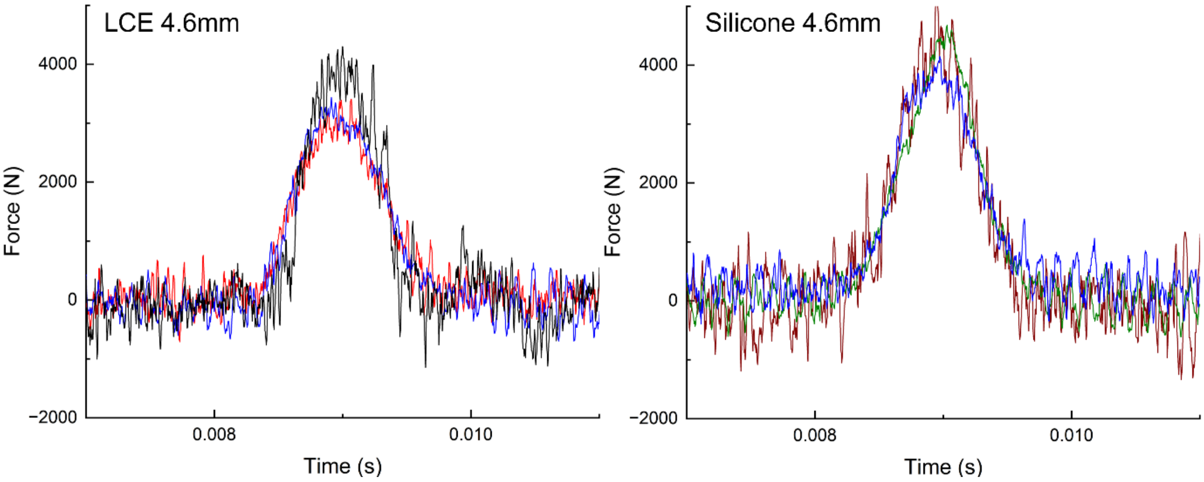

Supplement: Supplementary file 1 — Supplementary Information 1. [file 41598_2023_37215_MOESM1_ESM.pdf]
